# Ultrahigh Sensitivity of a Plasmonic Pressure Sensor with a Compact Size

**DOI:** 10.3390/nano11113147

**Published:** 2021-11-21

**Authors:** Chung-Ting Chou Chao, Yuan-Fong Chou Chau, Sy-Hann Chen, Hung Ji Huang, Chee Ming Lim, Muhammad Raziq Rahimi Kooh, Roshan Thotagamuge, Hai-Pang Chiang

**Affiliations:** 1 Department of Optoelectronics and Materials Technology, National Taiwan Ocean University, Keelung 20224, Taiwan; suyang191@gmail.com; 2 Centre for Advanced Material and Energy Sciences, Universiti Brunei Darussalam, Tungku Link, Gadong, Bandar Seri Begawan BE1410, Brunei; cheeming.lim@ubd.edu.bn (C.M.L.); chernyuan@hotmail.com (M.R.R.K.); roshan.kumara@ubd.edu.bn (R.T.); 3 Department of Electrophysics, National Chiayi University, Chiayi 600, Taiwan; shchen@mail.ncyu.edu.tw; 4 National Applied Research Laboratories, Taiwan Instrument Research Institute, Hsinchu 300, Taiwan; hjhuang@narlabs.org.tw

**Keywords:** metal-insulator-metal, pressure sensor, multiple modes, finite element method, nanophotonic

## Abstract

This study proposes a compact plasmonic metal-insulator-metal pressure sensor comprising a bus waveguide and a resonator, including one horizontal slot and several stubs. We calculate the transmittance spectrum and the electromagnetic field distribution using the finite element method. When the resonator’s top layer undergoes pressure, the resonance wavelength redshifts with increasing deformation, and their relation is nearly linear. The designed pressure sensor possesses the merits of ultrahigh sensitivity, multiple modes, and a simple structure. The maximum sensitivity and resonance wavelength shift can achieve 592.44 nm/MPa and 364 nm, respectively, which are the highest values to our knowledge. The obtained sensitivity shows 23.32 times compared to the highest one reported in the literature. The modeled design paves a promising path for applications in the nanophotonic field.

## 1. Introduction

Recently, surface plasmon polaritons (SPPs) are electromagnetic (EM) waves traveling along the dielectric-metal border and forming collective oscillations of electrons and photons at resonant wavelengths [1,2,3,4,5,6,7,8,9]. SPPs can facilitate light–matter interaction between dielectric medium and metal nanoparticles (MNPs) since they can configure light into the nanometer domain and overcome the diffraction limit [10,11,12,13,14,15]. SPPs based on metal-insulator-metal (MIM) bus waveguide (WG) combined with single or several nanocavities (or resonators) has a myriad of considerations due to its unique optical properties, including low losses, long propagation distance, easy fabrication, and compatible integrated optical circuits (IOCs) [16,17,18,19,20]. Plasmonic MIM WGs combined with resonators have many applications in nanophotonics fields [21,22,23], such as optical switches [24], beam splitters [25,26], perfect absorbers [27,28,29], optical filters [30,31,32], and plasmonic sensor [33,34,35] because of their robust light localization and confinement and light tunability at a subwavelength regime and excellent performance of sensitivity to the variation of ambient materials.

A MIM-based plasmonic pressure sensor is an optical element that can undergo exerted pressure and transform it into electrical or optical signals [19,36,37,38]. Plasmonic optical pressure sensors with the immune of EM wave disturbance have received considerable attention ever since numerous applications in the engineering field of biomedicines, mechanics, and electric optics [39]. Most reported optical pressure sensors are based on the Fabry–Perot interferometer [40,41], SiO_2_ diaphragm [42], monolithic capacitive pressure sensor [43], Mach–Zehnder interferometer [44], optical fibers [45,46], etc. They show a larger size and have a drawback of less compatibility to IOCs.

The absorption peaks of surface plasmon resonance (SPR) in gold nanospheres (AuNSs) and gold nanorods (Au NRs) are highly sensitive to their surrounding medium and to their refractive index changes. Martín-Sánchez et al. have found that the plasmonic properties of AuNSs can be used as the basis for sensing changes in the surrounding medium under very high pressure ranging from 0 to −60 GPa [47]. Runowski et al. reported that Au nanorods (NRs) could effectively detect phase transitions of compressed compounds, whether liquid and solid, by measuring their refractive index. They observed its subtle refractive index changes and the properties of high-pressure phases by using high-pressure absorption Vis-NIR spectroscopy [48].

In order to be compatible with IOCs, the strategy to miniaturize the pressure sensor’s size is crucial. Recently, many research groups proposed various optical pressure sensors to satisfy the compatibility of IOCs. Chaudhary et al. presented a hydrostatic pressure sensor using photonic crystal fibers (PCFs) with a sensitivity of 0.0116 nm/MPa [49]. Dinodiya et al. developed a photonic crystal’s pressure sensor based on Si and GaAs and claimed that the obtained sensitivity could reach 17.00 nm/GPa [50]. Another approach based on a nanostructure grating can detect pressure ganging in 0–1 kPa [51]. In ref. [37], Zhao et al. reported a nano-optomechanical pressure sensor employing a ring cavity with a sensitivity of 1.47 pm/kPa. Yao and his collaborators designed a fiber-tip pressure sensor, and the sensitivity can achieve 4.29 nm/MPa [40]. Wu et al. also illustrated a pressure sensor employing an H-type SPP resonator and found the measurable minimum pressure to be about 2.6 × 10^4^ Pa [52]. Yu et al. studied flexible plasmonic pressure sensors based on layered two-dimensional (2-D) heterostructures and reported the highest detecting sensitivity of 20.8 k/Pa at the SPR angle [53]. Recently, Tathfif et al. proposed a high sensitivity pressure sensor of 25.4 nm/MPa using thirty-four silver (Ag) nanorods embedded in the slot and bus WG and claimed that the obtained sensitivity is the highest one compared to the previous literature [54]. However, their designed structure has the drawback of complex fabrication because they place thirty-four Ag nanorods in bus WGs and resonators. Moreover, the sensitivity must further improve as high as possible in order to meet the current pressure sensing application requirements.

This paper designs a compact plasmonic pressure sensor composed of a MIM bus WG and a resonator, including a horizontal slot and several stubs. The resonator’s size of the designed sensor is as small as a few hundred nanometers. We employed the finite element method (FEM) to investigate the plasmonic pressure sensor’s transmittance features and EM wave distribution and calculated the relation between resonator deformation and sensitivity. Simulation results demonstrate that the resonance wavelength has a redshift when the resonator deforms. Thus, the resonator’s deformation can measure the sensitivity of the plasmonic pressure sensor.

Furthermore, the deformation is linearly proportional to the wavelength shift in a broad wavelength range. As a result, the designed pressure sensor has an ultra-high sensitivity of 592.44 nm/MPa, the highest value reported to date, and demonstrates 23.32 times the sensitivity compared to the highest one (i.e., ref. [54]) published before this study. Moreover, the designed structure is straightforward and is rarely investigated before. Due to high sensitivity and compactness, the proposed plasmonic pressure sensor is a pivotal candidate for different on-chip sensing purposes and offers a chance for designing a beneficial nanophotonic device.

## 2. Simulation Models, Analysis Method, and Fundamentals

**Figure 1:**
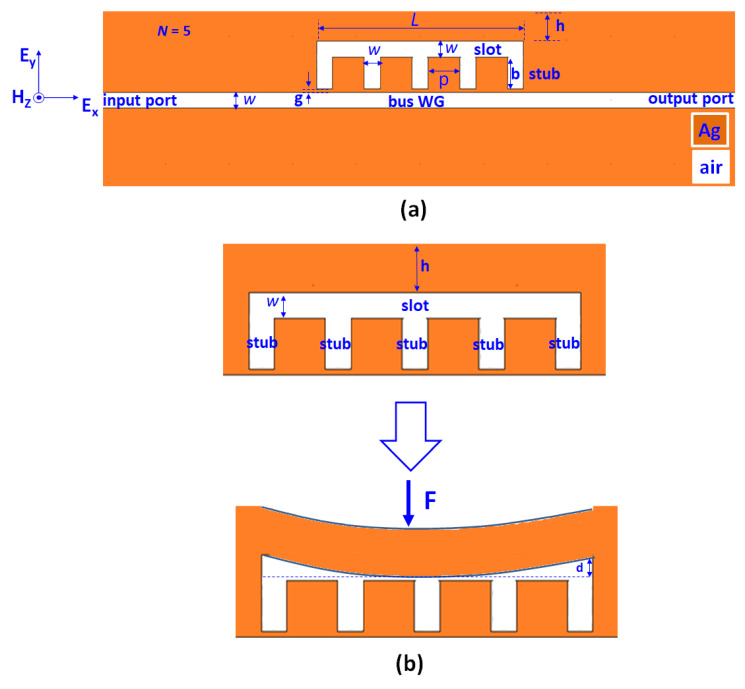
(**a**) Schematic diagram of the designed sensor. (**b**) Schematic diagram of an applied pressure *F* exerted on the Ag layer.

Figure 1a,b illustrate the schematic diagrams of the designed sensor, i.e., a MIM bus WG (width *w*) side-coupled to a resonator which contains one horizontal slot (width *w* and length *L*) and five vertical stubs (width *w*, height *b*, and gap distance between two stubs *P*), respectively. The thickness of the Ag layer above the horizontal slot is *h*, and the gap distance between the bus WG and the resonator is *g*. In Figure 1, the golden and white parts stand for Ag and air. If an applied pressure *F* is exerted on the top of the Ag layer, it will bend downwards with deformation of *d* (see Figure 1b), which is stated according to [37,52,54,55].

A TM-polarized incident light couples with the fundamental SPP mode [56,57,58] into the bus WG’s input port, and the transmission power reaches the output port [59,60]. The popular plasmonic metals are gold (Au) and Ag [61,62]. Ag is used in this study due to the cost consumption that must be considered for the future fabrication. Moreover, the EM wave response of Ag has the smallest imaginary part of relative permittivity within the near-infrared level. As a result, its power consumption is low compared to other metals (e.g., Au, copper, or platinum). The Drude model can characterize the permittivity (*ε*_m_) of Ag [63]. A 2-D physical model replaces the 3-D physical model because the structure height in the *z*-axis is much larger than the skin depth of SPPs in the x-axes and y-axes. FEM-based COMSOL Multiphysics with ultrafine mesh sizes was used to simulate the transmittance spectrum and EM wave distributions. Perfectly matched layers were used to absorb the outgoing waves without reflection. The SPPs’ mode can be generated in the plasmonic system when the incident light approaches the intrinsic resonance wavelength (λ_res_). If Δ*φ* = 2π*m* (*m* = 1,2,3 …), λ_res_ can be described as follows [64,65].

(1) $$\lambda_{\mathrm{res}}=\frac{2L_{eff}Re(n_{\mathrm{eff}})}{m-\frac{\phi }{2\pi }}{\;}_{}$$

Here, *L_eff_* represents the resonator’s effective length, *φ* and *Re(n*_eff_) are the phase shift and the real part of the effective refractive index, respectively, and *n*_eff_ is defined as follows:

(2) $$Re(n_{\mathrm{eff}})={\left(\varepsilon_{silver}+{\left(\frac{k_{}}{k_{0}}\right)}^{2}\right)}^{\frac{1}{2}}$$

where *k* = 2π/λ is the wave vector, and *k*_0_ is the wave vector in the free space.

The transmittance (T) can be calculated by T = *P_out_* (output power)/*P_in_* (input power). FWHM is the full width at half-maximum. Quantifying the ratio of the quality factor and modal volume indicates how sensitive the tailored device is.

High values of the QF/V ratio correspond to significant and robust light–matter interaction [66]. Therefore, the quality factor (QF) and modal volume can be defined as λ_res_/FWHM and QF/V, respectively [67,68,69]. 

**Figure 2:**
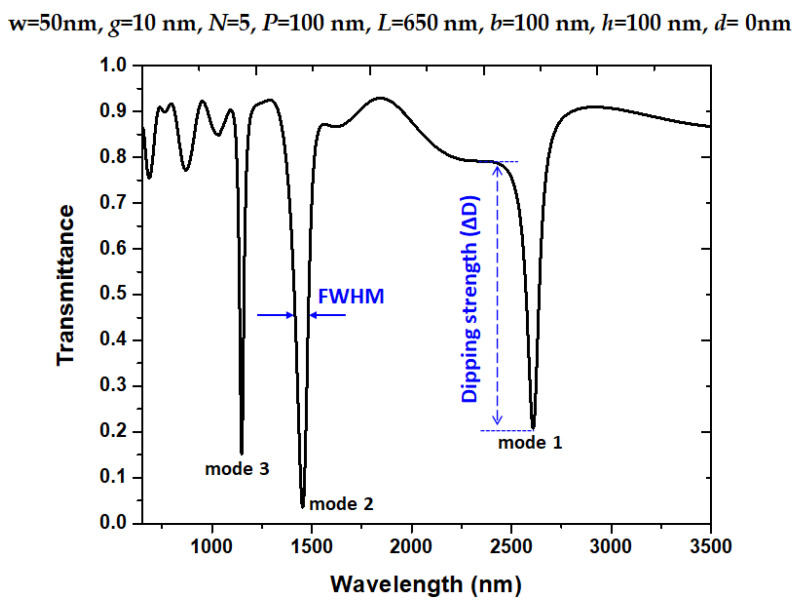
Transmittance spectrum for the designed structure without pressure on the system (i.e., *d* = 0 nm).

Moreover, we define the dipping strength (ΔD) in Equation (3), i.e., the difference between the transmittance peak (T_peak_) and transmittance dip (T_dip_) [70]; see the inset of Figure 2.

ΔD = (T_peak_ − T_dip_) × 100% (3)

Slater’s law can express the resonant frequency shift (*∂f*) and the variation in the resonator’s volume (*dV*) by using the following [71]:

(4) $$\frac{\partial f}{f}=-\frac{\left(\varepsilon_{0}E^{2}-\mu_{0}H^{2}\right)dV}{\int_{V}^{}\left(\varepsilon_{0}E^{2}+\mu_{0}H^{2}\right)dV}$$

where *f* is the resonant frequency of resonating area with a volume of *V*. *H* and *E* are magnetic and electric fields [72].

Regarding the top Ag layer as a flat plane (length *L* and thickness *h*), the pressure (*F*, in the unit of Pa) applied on the sensor and deformation (*d*) can be calculated as follows [52]:

(5) $$F=\frac{2Ydh^{3}}{L^{4}}$$

where *F* and *Y* represent the pressure applied on the sensor and Young’s modulus of Ag (*Y* = 7.5 × 10^10^ Pa), respectively. Wavelength shift (Δλ) is associated with the resonator’s deformation (*d*) with a coefficient of η. Therefore, Equation (5) can be modified as follows.

(6) $$F=\frac{2Yh^{3}}{\eta L^{4}}\Delta \lambda$$

The sensitivity (S) of the pressure sensor can be described by the following [55].

(7) $$S=\frac{\Delta \lambda }{\Delta F}=\frac{L^{4}\Delta \lambda }{2Yh^{3}\Delta d}$$

Measurement of transmission loss (TL) is a unit of decibel (dB), and it can utilize the following formula.

(8) $$TL=10\;log_{10}\frac{P_{in}}{P_{out}}\;(\mathrm{dB})$$

*P_in_* is the power of incident wave coming towards a defined area (i.e., the designed structure), and *P_out_* is the power of the wave transmitted away from the designed system.

Since the advance in nanofabrication, the fabrication of the designed pressure sensor is achievable with current manufacturing technologies [73]. The MIM waveguide with a rectangular shape can be implemented by using stripping and ion beam lithography processes [74]. However, the objective of this paper is not to concentrate on fabrication methods. As an alternative, several potential studies that studied this issue closely is suggested [75,76].

## 3. Results and Discussion

Figure 2 illustrates the transmittance spectrum for the designed structure at the situation without any pressure on the system (i.e., *d* = 0 nm). We retain bus WG’s width as *w* = 50 nm to guarantee that the TM mode can travel in bus WG, horizontal slot and bottom stubs and *g* = 10 nm was used for obtaining a better coupling effect between bus WG and resonator. The default structural parameters, *w*, *g*, *P*, *L*, *b*, *h*, and *d*, are 50 nm, 10 nm, 100 nm, 650 nm, 100 nm, and 0 nm, respectively. The resonator size of the designed structure is compact and much smaller than previous designs (e.g., [77,78]). We marked the mode numbers in Figure 2. As observed, three resonance modes correspond to mode 1 and to mode 3, which are attributed to the cavity plasmon resonance (CPR) and SPR modes between bus WG and resonator [10,79]. We compared the resonance wavelength (λ_res_), FWHM, ΔD, and QF of the designed structure at corresponding resonance modes in Table 1. According to Table 1, we found that resonance dips in the developed system have a more profound dipping strength (∆D), a narrower FWHM, and a higher QF than others in the published literature (e.g., [52,54,80]). Moreover, the calculated transmission losses (based on Equation (8)) are 8.34 dB, 11.61 dB and 9.13 dB, respectively. According to the simulations (not shown here for the sake of simplicity), transmission loss has less influence on sensing performance since the designed device has low transmission loss values at resonance modes. Based on ref. [66], the mode volumes (QF/V) are 0.224 (λ/*n*)^−3^, 0.507 (λ/*n*)^−3^, and 0.509 (λ/*n*)^−3^ for modes 1–3, respectively, where λ (i.e., λ_res_) and *n* denote resonance wavelength and refractive index in the resonator. For practical fabrication, any height larger than 800 nm will agree with the simulations [54,81]. Therefore, we use the height of 900 nm in order to calculate the volume (i.e., 650 nm × 100 nm × 900 nm in this case) for mode volume (QF/V).

**Figure 3:**
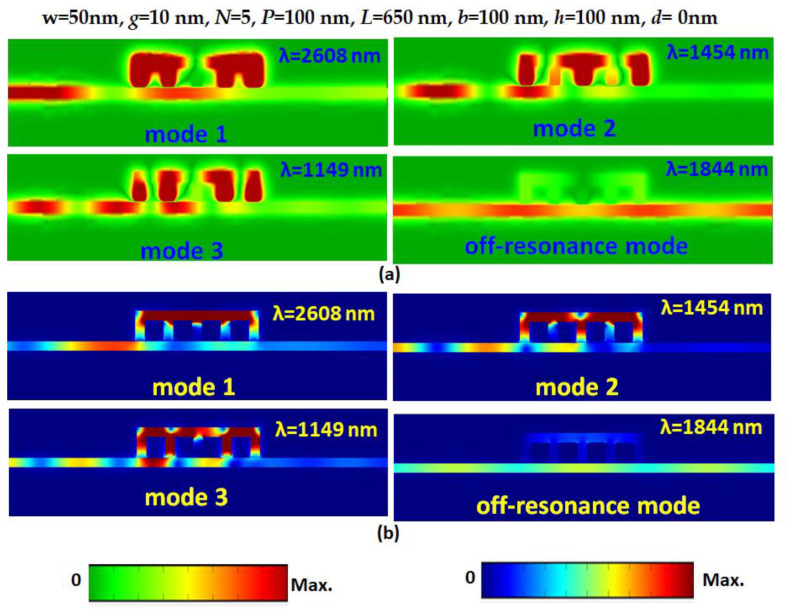
Normalized (**a**) magnetic field intensity and (**b**) electric field intensity at the corresponding resonance modes and one of the off-resonance modes.

**Figure 4:**
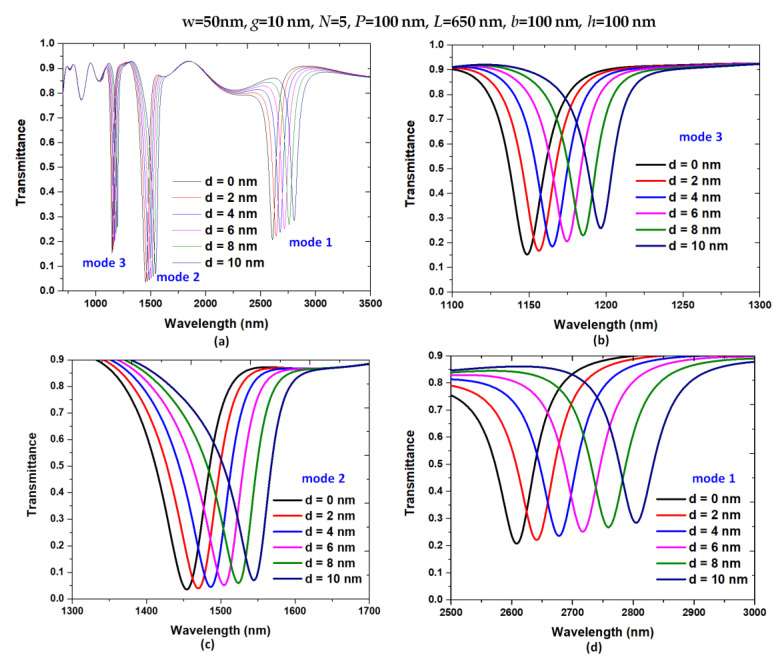
Transmittance spectra of the designed structure with a variation of *d* from 0 to 10 nm with an interval of 2 nm in the wavelength of (**a**) 700–3500 nm for modes 1–3, (**b**) 1100–1300 nm for mode 3, (**c**) 1300–1700 nm for mode 2, and (**d**) 2500–3000 nm for mode 1, respectively.

Figure 3a,b illustrate the normalized magnetic field intensity and electric field intensity at the corresponding resonance modes and one of the off-resonance modes in order to observe its physical nature. As observed, |H| and |E| fields are effectively confined in the resonator due to the constructive interference between bus WG and the resonator, showing remarkable CPR effect and excellent light–matter interaction. The dominant |H| and |E| fields are spread in the horizontal slot and bottom stubs at resonance modes. Note that most of the |H| and |E| fields localize within the horizontal slot where the pressure can be exerted. Thus, the intensity order of |H| and |E| fields is mode 1 > mode 2 > mode 3 in the horizontal slot. This implies that pressure sensitivity in mode 1 is higher than the other modes, and we can verify this phenomenon in Figure 4. In contrast, the |H| and |E| fields are hardly trapped in the horizontal slot and bottom stubs at off-resonance modes due to the destructive interference between bus WG and resonator.

**Figure 5:**
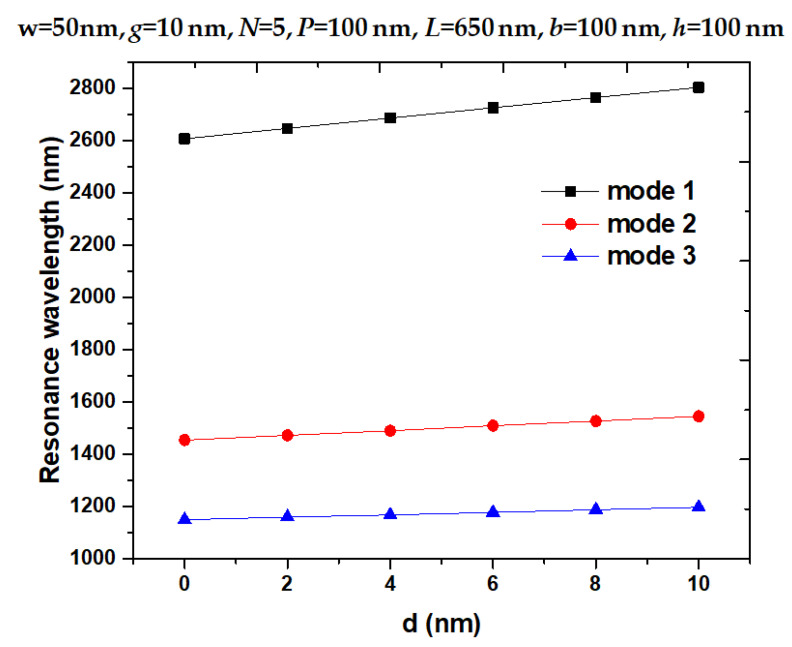
Resonance wavelength versus variation of *d* from 0 to 10 nm with an interval of 2 nm.

Pressure influence on the upper horizontal slot denotes deformation [37,52,54,55]. Figure 4a–d show the transmittance spectra of the designed structure with a variation of *d* from 0 to 10 nm with an interval of 2 nm in the wavelength of 700–3500 nm for modes 1–3, 1100–1300 nm for mode 3, 1300–1700 nm for mode 2, and 2500–3000 nm for mode 1, respectively. Referring to Figure 3, most |H| and |E| fields are trapped in the upper horizontal slot where the pressure is applied. Therefore, the deformation of the upper horizontal slot will reduce the air space in the horizontal slot and increase the pressure in its space (see Figure 1b). By raising *d*, λ_res_ undergoes a redshift since the lessening horizontal slot’s volume enhances the CPR effect, which results in good agreement with Equations (4)–(7). Therefore, it can expect that λ_res_ redshifts with the increase in *d*, the resonant wavelength moves toward a more extended wavelength region. As observed in Figure 4b–d, the resonance wavelength shifts (∆λ_res_) are 197 nm, 91 nm, and 48 nm for modes 1–3, and the corresponding sensitivity (*S*) calculated using Equation (7) can reach 23.44, 10.82 and 5.71 nm/MPa for modes 1–3, respectively. Moreover, we showed the λ_res_ versus variation of *d* from 0 to 10 nm with an interval of 2 nm in Figure 5 and found a linear relationship between them.

**Figure 6:**
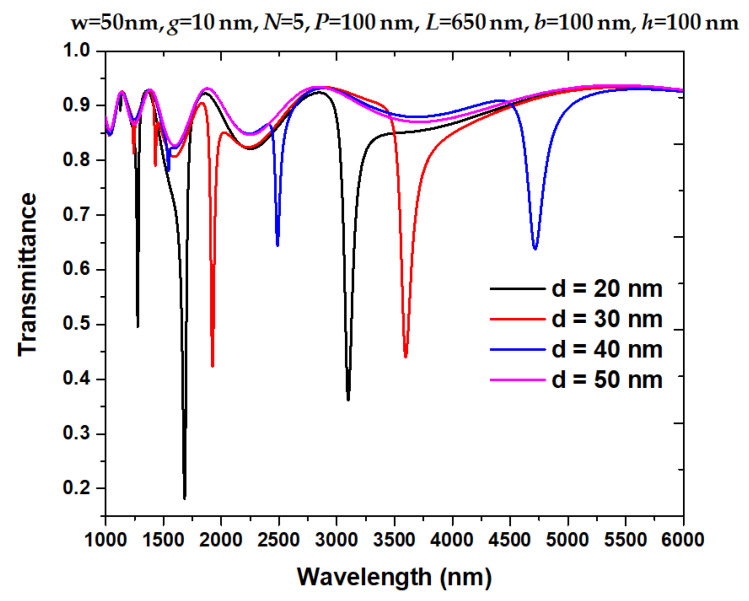
Transmittance spectrum of the proposed structure when deformation *d* = 20 nm, 30 nm, 40 nm, and 50 nm, respectively.

Deformation *d* is in the range of 0 < *d* < 50 nm because of the slot height *w* = 50 nm. Figure 6 shows the transmittance spectrum of the proposed structure when deformation *d* = 20 nm, 30 nm, 40 nm, and 50 nm, respectively. As observed in Figure 6, the transmittance dip will redshift as *d* increases and vanish when *d* = 50 nm. The redshift is in line with Equation (4) (i.e., Slater’s law), and the vanishment of transmittance dip is a result of the slot’s volume approaching zero when *d* = 50 nm. Moreover, FWHM will enlarge, and the dipping strength will reduce when *d* increases from 20 nm to 40 nm. As a result, asymmetricity will affect the linearity of the resonant wavelength vs. *d* if *d* ≥ 50 nm.

**Figure 7:**
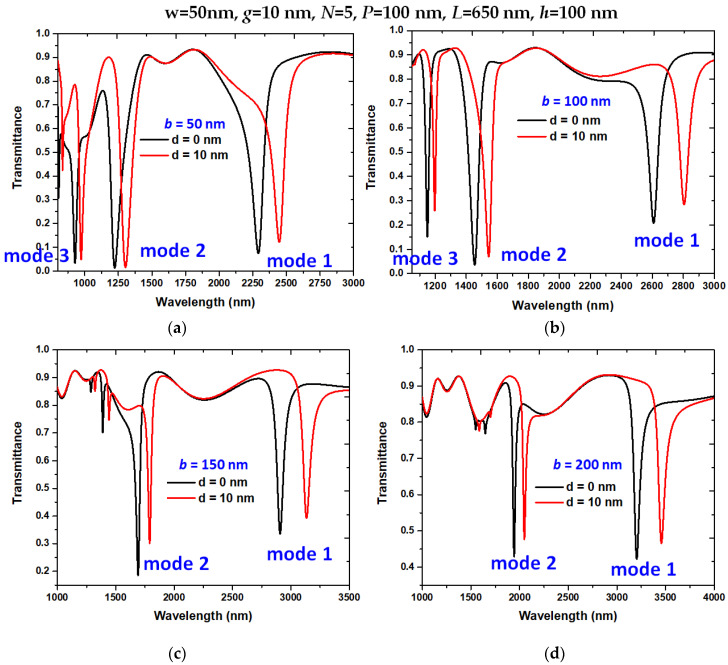
Transmittance spectra of the designed structure with variation of the height of stubs (*b*), (**a**) *b* = 50 nm, (**b**) *b* = 100 nm, (**c**) *b* = 150 nm, and (**d**) *b* = 200 nm, respectively.

The structural parameters have a great impact on the sensing performance of the designed plasmonic pressure sensor. As mentioned, we set *w* = 50 nm for the width of bus WG, slot, and stub to assure that the TM mode can couple in the optical path of the plasmonic system and set g = 10 nm for efficient coupling between bus WG and resonator. Thus, we inspected the other two structural parameters, i.e., *b* and *N*, while retaining the other structural parameters. First, we investigated the influence of the stub’s height, *b*, on the transmittance spectrum, as shown in Figure 7a–d, respectively. As observed, the transmittance dips redshifts with increasing *b* and *d*, while ∆D reduces with raising *b* due to the transmission loss in the resonator. According to Figure 7a–d, we compared the resonance wavelength shift (∆λ_res_) and sensitivity S(nm/MPa) of the designed structure for *b* = 50, 100, 150, and 200 nm in Table 2. Note that resonance wavelength shift and sensitivity will increase with increasing *b*. The maximum ∆λ_res_ and S(nm/MPa) in modes 1 can reach (254 nm, 30.23 nm/MPa) and show values 83.21% higher than those found in a previous study [55]. It implies that if we extend the length of the stub, it can anticipate a higher (∆λ_res_, S). For example, we can attain (∆λ_res_, S) in mode 1 and mode 2 of (276 nm, 32.85 nm/MPa) and (109 nm, 12.57 nm/MPa) when *b* = 250 nm (not shown here for simplicity). However, the improved (∆λ_res_, S) by lengthening *b* will enlarge the device’s size, raising the Ohmic loss in the designed system and negatively influencing the miniaturization of IOCs.

**Figure 8:**
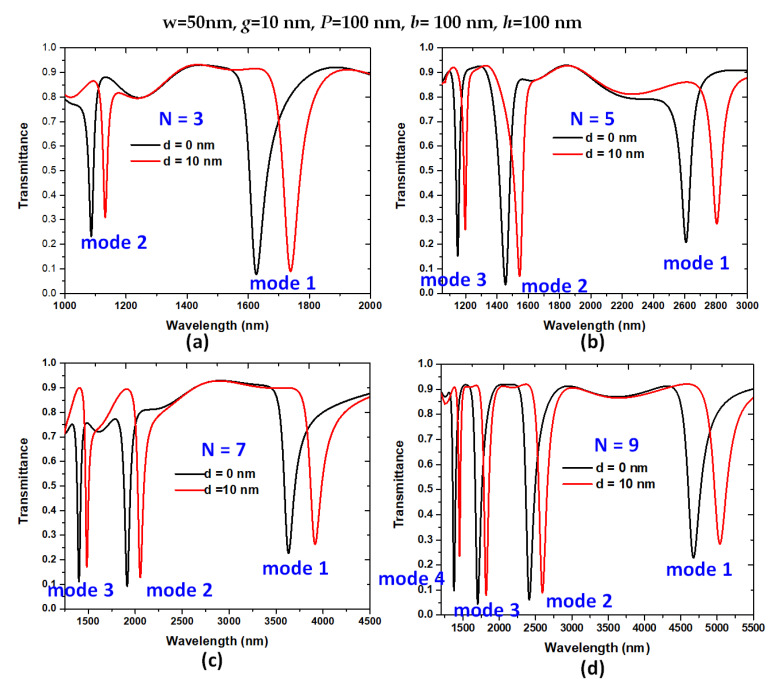
Transmittance spectra of the designed structure with a variation of the number of stubs (*N*), (**a**) *N* = 3, (**b**) *N* = 5, (**c**) *N* = 7, and (**d**) *N* = 9, respectively.

Figure 8a–d depict the transmittance spectra of the designed structure with a variation of the number of stubs (*N*), when *N* = 3, 5, 7, and 9, respectively. In these cases, the corresponding length of horizontal slot (*L*) is 350 nm, 650 nm, 950 nm, and 1250 nm, respectively. In Equation (7), the structural parameter *L* is positively proportional to sensitivity (S), which means that a more significant stub number (*N*) will receive higher pressure sensitivity. As observed in Figure 8a–d, the resonance wavelength and the number of modes increases with increasing *N*. We summarized (∆λ_res_, S) of the designed structure for *N* = 3, 5, 7, and 9 in Table 3. Based on Figure 8 and Table 3, (∆λ_res_, S) reveals a significant advance with increasing *N*, e.g., ∆λ_res_, and *S* in mode 1 achieved 364 nm and 592.44 nm/MPa when *N* = 9. However, a more significant *N* representing the greater number of stubs could enlarge the resonator’s size and increase Ohmic loss in bus WG and resonator. These values obtained from Table 3 are much higher than those of reported articles. We compare the designed plasmonic pressure sensor with the published designs in Table 4. The designed structure displays the most heightened sensitivity (≈23.32 times) than compared to [50] to the best of our knowledge. In addition, the pressure (in the unit of Pa) applied to the designed sensor can be calculated based on the Equations (5) and (7). For example, when the parameters of (*w*, *g*, *N*, *P*, *L*, *h*) = (50 nm, 10 nm, 97, 100 nm, 650 nm, 100 nm), the measurable minimum pressures are about 8.404 Pa, 8.410 Pa, and 8.406 Pa for modes 1, 2 and 3, respectively.

## 4. Conclusions

This study proposes a compact and simple plasmonic pressure sensor structure comprising a bus waveguide and a resonator with one horizontal slot and several stubs. The finite element method is employed to calculate the transmittance spectrum and the electromagnetic field distribution of the designed structure. The transmittance spectrum reveals a redshift for increasing the resonator’s deformation. The designed pressure sensor can apply to some situations for small-scale ultrahigh-pressure sensitivity and is compatible with IOCs. The result shows that the resonance wavelength redshift has a linear relationship with the resonator’s deformation. Furthermore, we found that (∆λ_res_, S) reveals a significant advance with the rising number of stubs. The designed pressure sensor has an ultra-high sensitivity of 592.44 nm/MPa, which is the highest value to our knowledge and achieves 23.32 times the sensitivity compared to the highest value in the literature. Thus, the designed plasmonic pressure sensor paves a path for pivotal applications used in nanophotonic devices.

## Figures and Tables

**Table 1:** Comparison of λ_res_, FWHM, ΔD, and QF of the designed structures at corresponding resonance modes.

	Mode 1	Mode 2	Mode 3
λ_res_ (nm)	2608	1454	1149
FWHM (nm)	60.00	50.00	40.00
ΔD (%)	58.37	92.16	90.24
QF	43.47	29.08	36.35

**Table 2:** Comparison of (∆λ_res_(nm), S(nm/MPa)) of the designed structure for *b* = 50, 100, 150, and 200 nm.

(∆λ_res_, S)	Mode 1	Mode 2	Mode 3
*b* = 50 nm	(155, 18.45)	(80, 9.25)	(45, 5.34)
*b* = 100 nm	(197, 23.44)	(91, 10.82)	(48, 5.71)
*b* = 150 nm	(227, 27.01)	(99, 11.78)	
*b* = 200 nm	(254, 30.23)	(105, 12.50)	

**Table 3:** Comparison of (∆λ_res_(nm), S(nm/MPa)) of the designed structure for *N* = 3, 5, 7, and 9.

(∆λ_res_, S)	Mode 1	Mode 2	Mode 3	Mode 4
*N* = 3	(113, 11.31)	(46, 4.61)		
*N* = 5	(197, 23.44)	(91, 10.82)	(48, 5.71)	
*N* = 7	(283, 153.67)	(139, 73.85)	(85, 46.16)	
*N* = 9	(364, 592.44)	(181, 294.60)	(116, 188.80)	(74, 120.44)

**Table 4:** Comparison of the designed plasmonic pressure sensor with the published designs.

Reference/Year	Structure/Size	Max. S (nm/MPa)	Max. ∆λres (nm)	Operating Wavelength
[82]/2008	long PM-PCF/58.4 cm	3.42	5.30	1550 nm < λ < 1555 nm
[37]/2012	nanoring resonator/1500 × 1500 μm^2^	1.47	-	1602.3 nm < λ < 1602.9 nm
[80]/2016	π-shaped resonator/400 × 150 nm^2^	8.5	80.00	600 nm < λ < 1800 nm
[55]/2018	double square resonator/700 × 500 nm^2^	16.5	103.00	350 nm < λ < 1350 nm
[46]/2020	thin-walled oval cylinder/6 × 17 × 0.5 mm^3^	1.198	-	1549 nm < λ < 1558 nm
[54]/2021	34 Ag nanorods in slots/800 × 230 nm^2^	25.4	92.93	1400 nm < λ < 2200 nm
This work	one slot and nine stubs/1250 × 150 nm^2^	592.44	364.00	1000 nm < λ < 5500 nm

## Data Availability

No data available.
